# Developing a response to family violence in primary health care: the New Zealand experience

**DOI:** 10.1186/s12875-016-0508-x

**Published:** 2016-08-20

**Authors:** Claire Gear, Jane Koziol-McLain, Denise Wilson, Faye Clark

**Affiliations:** 1 Centre for Interdisciplinary Trauma Research, School of Clinical Sciences, Faculty of Health and Environmental Sciences, Auckland University of Technology, Private Bag 92006, Auckland, 1142 New Zealand; 2 School of Public Health & Psychosocial Studies, Auckland University of Technology, Auckland, New Zealand; 3 Doctors for Sexual Abuse Care Inc., Auckland, New Zealand

**Keywords:** Program evaluation, Domestic violence, New Zealand, Program sustainability, Primary health care, Complex adaptive system (non-MESH)

## Abstract

**Background:**

Despite primary health care being recognised as an ideal setting to effectively respond to those experiencing family violence, responses are not widely integrated as part of routine health care. A lack of evidence testing models and approaches for health sector integration, alongside challenges of transferability and sustainability, means the best approach in responding to family violence is still unknown. The Primary Health Care Family Violence Responsiveness Evaluation Tool was developed as a guide to implement a formal systems-led response to family violence within New Zealand primary health care settings. Given the difficulties integrating effective, sustainable responses to family violence, we share the experience of primary health care sites that embarked on developing a response to family violence, presenting the enablers, barriers and resources required to maintain, progress and sustain family violence response development.

**Methods:**

In this qualitative descriptive study data were collected from two sources. Firstly semi-structured focus group interviews were conducted during 24-month follow-up evaluation visits of primary health care sites to capture the enablers, barriers and resources required to maintain, progress and sustain a response to family violence. Secondly the outcomes of a group activity to identify response development barriers and implementation strategies were recorded during a network meeting of primary health care professionals interested in family violence prevention and intervention; findings were triangulated across the two data sources.

**Results:**

Four sites, representing three PHOs and four general practices participated in the focus group interviews; 35 delegates from across New Zealand attended the network meeting representing a wider perspective on family violence response development within primary health care. Enablers and barriers to developing a family violence response were identified across four themes: ‘Getting started’, ‘Building effective relationships’, ‘Sourcing funding’ and ‘Shaping a national approach to family violence’.

**Conclusions:**

The strong commitment of key people dedicated to addressing family violence is essential for response sustainability and would be strengthened by prioritising family violence response as a national health target with dedicated resourcing. Further analysis of the health care system as a complex adaptive system may provide insight into effective approaches to response development and health system integration.

## Background

Health care professionals are uniquely placed to deliver services for victims of family violence, particularly within primary health care settings [1–6]. However, the health care sector has been slow in delivering a response to family violence and contributing to wider multi-sectoral efforts [3, 4]. Current international guidelines strongly recommend health care professionals offer immediate first-line support to women who disclose any form of violence or sexual violence by an intimate partner, including facilitating disclosure, offering support and referral, providing medical treatment and follow-up care, and documenting evidence [3, 4, 7]. It is also strongly recommended service delivery be prioritised for primary health care [4], a setting recognised as a victim’s first or for many, the only point of contact with health care professionals [6]. Health care professionals should understand the relationship between exposure to violence and poor health and be able to respond appropriately [8].

A comprehensive systems approach to service delivery alongside practice guidelines has been advocated to support a programme of sustainable family violence identification and intervention within health care settings [5, 9, 10]. Yet a lack of evidence testing different models and approaches for health care sector integration means the best approach for responding to family violence is still unknown. It is not surprising then, that current responses vary significantly across health care systems internationally [3, 11]. Issues of transferability and sustainability across health care systems and settings continue to present challenges, regardless of the approach employed [2, 11]. Consideration of the organisational context in which a new intervention will be embedded is important to identify factors which will challenge or promote the implementation and sustainability of the intervention [12].

In New Zealand, primary health care services (such as general practitioners and practice nurses) are largely delivered by private general practices who receive public funding from the Ministry of Health, distributed by their District Health Board (DHB) through their regional Primary Health Organisation (PHO) [13]. Currently, District Health Boards are implementing Ministry of Health directed Violence Intervention Programmes (VIP) within hospital-based care [10], but there is no strategy to inform family violence responsiveness within primary health care settings [5]. Practice resources are available to inform primary health care professionals in responding to intimate partner violence [14] and child abuse [15]. In addition, the Ministry of Health, alongside the New Zealand Police and the Accident Compensation Corporation, contribute funding to the voluntary professional medical body Doctors for Sexual Abuse Care (DSAC). DSAC provide medical response to abuse and sexual assault training courses and an accreditation programme for volunteer general practitioners (GPs) and other primary health care professionals [16].

In 2012, the Ministry of Health commissioned the development of the Primary Health Care Family Violence Responsiveness Evaluation Tool (‘the Tool’) [5] to complement existing Partner Abuse and Child Abuse and Neglect Delphi Tools [10, 17] used to evaluate hospital-based VIP. Twenty-nine expert panellists, representing diverse family violence prevention and intervention organisations across New Zealand, participated in a modified Delphi method to identify ideal primary health care family violence response indicators. The final Tool encompassed 143 indicators within 10 categories organised to guide response development (i.e. from Governance & Leadership to Quality Improvement). Subsequently pilot tested within six primary health care sites, the phased approach of the Tool showed promise for supporting effective, sustainable programme implementation [5]. The Tool was made available to guide primary health care settings in implementing a formal systems-led response, but it did not coincide with a formal sector strategy to establish a national primary health care system response to family violence and was not supported by further knowledge needed to support integration within practice. Given the limited understanding of realising a primary health care response to family violence, both in New Zealand and internationally, we aimed to capture the qualitative experience of developing a response to family violence within New Zealand primary health care settings. Specifically, we collected data to identify the enablers, barriers and resources required to maintain, progress and sustain a family violence response.

## Methods

In this paper we present qualitative descriptive data from two methods of data collection: follow-up evaluation focus group interviews and the outcomes of a primary health care family violence network group activity.

### Follow-up evaluation focus group interviews

The six selected primary health care sites (inclusive of a PHO and affiliated general practice) who participated in the pilot testing of the Tool [5] were invited to participate in a 24 month follow-up evaluation from March to June 2012 to capture response development progress. Participation was on a voluntary basis and sites were excluded if they felt unable to participate. The follow-up evaluation aimed to support the continued development of a family violence response by (a) partnering with DSAC to provide consultation on practical issues, and (b) nurturing a collaborative working relationship with sites. Consent to participate was obtained from senior management at both the PHO and general practice who then appointed representatives to serve as follow-up evaluation liaisons. Researcher (CG) worked in collaboration with liaisons to prepare for the evaluation, providing evaluation documents (including The Tool) and answering queries.

The follow-up evaluation was designed to begin with a 30-min semi-structured focus group interview which aimed to capture the enablers, barriers and resources required to maintain, progress and sustain a response to family violence. Follow-up evaluation liaisons were responsible for coordinating the participation of relevant people involved in the site family violence response. Focus group interviews were led by the researcher (CG; experienced in evaluation methods), in partnership with a DSAC consultant (experienced in medical responses to abuse and sexual assault), directed by an interview guide of eight open-ended questions. The DSAC consultant was provided with information on qualitative interviewing in medical settings prior to the follow-up evaluation to support quality data collection.

The interview was designed to allow participants to speak freely about their experiences in their context and discuss challenges and opportunities. For these reasons, focus group interviews were designed to be conducted onsite at both the general practice and PHO, although some sites chose to convene a focus group inclusive of both. Ethics approval was extended by the New Zealand Health and Disability Ethics Committee (CEN/09/09/060) for the follow-up evaluation following additional provisions for tape-recording participants during the focus group interviews. Written consent to tape-record was obtained from PHO and general practice senior management upon agreeing to participate in the follow-up evaluation and verbally reconfirmed with participants prior to beginning the focus group interview. Focus group transcripts were de-identified and securely stored electronically using participant code numbers.

### Primary health care family violence network group activity

In addition to the development of the Tool [5] and the follow-up evaluation, growing sector interest generated a New Zealand primary health care family violence response network event (the Network) at the Auckland University of Technology in May 2012, sponsored by the Ministry of Health. The event was advertised for those interested or active in primary health care family violence prevention and intervention across New Zealand to join with others working to address family violence as a primary health care issue. The event programme, developed by a working group of researchers (CG & JKM), PHO family violence coordinators and a DSAC representative, included exemplars of primary health care family violence response development from five locations, provided resources to support response development and developed recommendations to inform national response development. The programme also included an activity based on the world café method, in which groups of delegates were asked to identify response development barriers and implementation strategies using the Tool [5] categories and indicators as prompts (led by JKM; experienced in health system and violence against women research methods). Following sharing and additional input from all groups, the final outcomes of this activity were recorded (written) and collated for analysis. Data collection served the Network event purpose of supporting response development; no identifying information was captured. Participation in all event sessions were voluntary and delegates could choose not to participate at any time.

### Analysis

Focus group interviews were tape-recorded, and along with the results of the national primary health care network event session, transcribed verbatim. As both sources addressed the core research question, data were triangulated across the two methods of data collection and examined thematically using a qualitative descriptive perspective following Braun and Clarke’s six steps of thematic analysis [18], with data management supported by the use of NVivo (v.10). Analysis was conducted inductively to avoid preconceived ideas and to explore how a response was developed; the process was iterative and reflexive. To increase reliability, codes were negotiated by four core research team members (CG, JKM, DW, FC) on a sample of the data prior to continued coding by the first author (CG). Emergent themes were reviewed by two core research team members (CG & JKM) for content validity.

## Results

Four sites, representing three PHOs and four general practices located across the urban North Island of New Zealand consented to participate in a focus group interview. A total of 11 participants in focus groups ranging from one to two were interviewed, including three Family Violence Intervention Coordinators, one Practice Nurse, one Social Worker, one General Practitioner, one Clinical Facilitator, two General Practice Managers, and two PHO Health Promotion Managers. Thirty-five delegates attended the Network event included GPs, nurses, academics and representatives from DHBs, PHOs, the Ministry of Social Development, DSAC and violence prevention non-government organisations (supported by government and/or community funding) who provided a wider perspective on family violence response development within primary health care.

Data supported four themes: ‘Getting started’, ‘Building effective relationships’, ‘Sourcing funding’ and ‘Shaping a national approach to family violence’. The following provides a description and exemplars of response development enablers, barriers and resources. Table 1 provides a list of family violence response development enablers across the four themes, which in turn also highlights the gap in knowledge regarding how to achieve this improved response.

**Table 1:** Family violence response development enablers

Local	*Getting started*
	Appoint a Key Resource Person supported by a family violence response steering group and team of champions
	Establish a consultation pathway to a family violence specialist to provide expert advocacy and address capacity issues
	*Building relationships*
	Engage strong management and clinical leadership support early in response development
	Ensure response development is up-to-date to maintain health professional confidence in response
	*Shaping a national response to family violence*
	Develop an autonomous response which meets local context and population needs
Community	*Building relationships*
	Establish strong community relationships, share information and knowledge and generate enthusiasm for developing a comprehensive quality response
	Support relationships by encouraging attendance at family violence response group meetings, sending newsletters, establishing information pathways, visiting general practices with specialists
National	*Getting started*
	Prioritise family violence as a health issue for primary health care
	*Sourcing funding*
	Evidence high-level organisational support by providing dedicated funding
	*Shaping a national response to family violence*
	Prioritise family violence as a target health issue and provide support to implement a comprehensive quality response
	Coordinate a national health care approach to family violence which allows for local autonomy
	Consider different implementation strategies for different levels of health care (primary, secondary, tertiary)

### Getting started

A response to family violence began when a site recognised a need to support those experiencing family violence. Sites then considered it necessary to appoint a Key Resource Person to lead and coordinate the response, ideally supported by a steering group and team of champions. Initially, the Key Resource Person would provide strategic direction, find local expertise, collaborate and share information with others and supply up-to-date resources. As the response progressed the Key Resource Person would lead the development of a strategic plan, family violence intervention policy and an advanced documentation form, engage with clinical leadership, local affiliated health providers and the local community, cultivate referral pathways and organise education opportunities. This role was crucial to the sustainability of the response and required strong management and clinical leadership support.

A major challenge to response development was a perceived lack of mandate to address family violence as a health issue. A sector focus on improving Ministry of Health PHO Performance Programme (PPP) health targets (such as smoking cessation) meant PHO and general practice resources were channelled to achieve those targets, causing concern for the continuation of other initiatives. “But because it [the family violence response] is not a PPP target, it’s in jeopardy and it’s not seen as a priority” (Family Violence Intervention Coordinator and PHO Health Promotion Manager). Both focus group and Network participants identified the government’s focus on achieving health targets as disabling when introducing a new innovation such as family violence prevention and intervention. To support response development, participants considered it essential for government to prioritise family violence as a health issue alongside dedicated funding.

*Well actually what would be greater than money is…to have that whole of organisation approach…we’ve currently got smokefree as a health target so there’s huge shift…pressure comes to bear from the DHB on to the PHO…to the whole organisation. So the whole capacity of the organisation is then shifted, funding is made available, people are encouraged, this is what needs to happen for the target to be met and I can tell you in the space of months the difference is phenomenal* (PHO Health Promotion Manager).

Competing priorities and capacity issues often stalled response development. Sites frequently reported being “overloaded”. “Oh yeah just a workload challenge…cause family violence is only one part of general practice. It’s one corner of it […], we’re not called general practice for nothing you know” (Practice Manager). Within three evaluation sites, an established consultation pathway to a family violence specialist helped address capacity issues and sustain overall response development. This role assisted clinicians following a positive disclosure, provided victims with expert advocacy and helped to increase overall capacity by freeing up clinician time. The role was implemented in different ways including: (a) access to a community based advocate for phone consultations who could visit practices when required, (b) an onsite social worker responsible for social issues, and (c) a team of practice champions that clinicians and nurses were able to call or visit.

[When] *they get a positive disclosure they don’t know what to do with there’s someone there at the end of the phone or they can get off their bottom and go for a walk and find someone that will help them* (Family Violence Intervention Coordinator).

### Building effective relationships

Early engagement with clinical leadership within the organisation was essential in gaining response traction. *“*If you want to get GPs to change their practice you have to get GPs to persuade them to do it” (Family Violence Intervention Coordinator). Keeping the response development up-to-date, such as keeping policies and advanced documentation form aligned with current guidelines, served to maintain clinicians and other health professionals’ confidence in the response. “You have policies that need to be renewed every 2 to 3 years […] So you need to have those consistently updated so no one loses trust in what is being put out there already” (Clinical Facilitator).

**Fig. 1:**
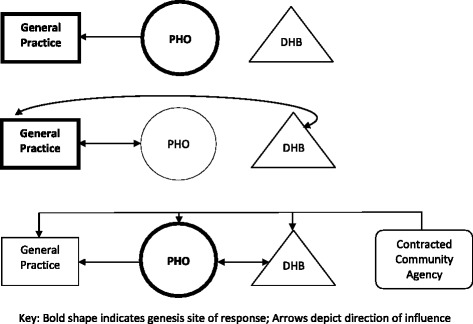
Site relationships

Primary health care practice relationships with regional PHOs and DHB secondary and tertiary health care services varied (see Fig. 1). None of the evaluation sites had yet developed a comprehensive approach for family violence response across local services and in some cases, there was a perceived reluctance to work together. At the time of data collection DHBs were implementing a Ministry of Health directed Violence Intervention Programme (VIP) without a mandate to include primary health care, general practices had established independent family violence policies and community relationships, and PHOs were piloting a systems approach to family violence to be implemented within general practices, independent of DHBs. This variability meant under-resourced, “overloaded” general practices were generally unwilling to become involved in a response which was not Ministry of Health directed and supported.

Nevertheless, Key Resource Persons developed relationships by encouraging attendance at steering group meetings, sending newsletters, establishing information pathways between organisations and visiting general practices with other specialists.

*…and that’s why in the end we asked our specialist to come with us, so she can see for herself that it* [primary health care] *is a different beast, and well actually it turned our relationship around … and so I believe that the nurses really engaged with the GP and said this is part of our business and the whole practice came to the training, all their non-clinicians as well* (Family Violence Intervention Coordinator).

Strong relationships with specialist community family violence service agencies were also essential in supporting response development. Regular family violence response steering group meetings provided space to share information and knowledge with other local health and social service providers and generate enthusiasm to return to their respective organisations. However, the lack of a comprehensive approach across localities meant community service representatives were asked to attend a number of meetings at different health locations creating a strain on agencies and the overall response to family violence for the local population.

### Sourcing funding

Funding the development of a family violence response was perceived as indicative of high-level organisational support. Three primary health care sites had successfully procured funding to support their programme development. The source of funding varied (e.g. a DHB contract, a medical research foundation grant, a Ministry of Health innovation grant and a Ministry of Social Development community contract); all funding sources were fixed term which significantly impacted on response progress and sustainability. The loss of funding threatened the loss of the Key Resource Person, a role recognised as crucial to response sustainability.

*But then there won’t be the support, for it* [the response] *going forward, and that really concerns me. When there’s been instances where people are screened inappropriately, and think that it’s ok, but it’s that lack of training, it’s that lack of knowledge, it’s that lack of an update…that’s not right. You’re going to endanger someone* (Family Violence Intervention Coordinator).

Fixed-term funding also created fatigue in justifying funding at the end of each fixed term and hampered organisations’ ability to contemplate sustainable service planning. Difficulties in measuring and obtaining evidence of the response process and impact meant it was difficult to demonstrate its merits. “That has been an ongoing struggle, is actually how do you report this stuff? We know that it works, but how do you show that it works?” (General Practice Manager and Social Worker). Both sites and the Network also raised concerns about how potential future funding would be distributed across health providers and how to avoid funding duplication and silos.

### Shaping a national response to family violence

At the time of data collection, a number of different family violence prevention and intervention initiatives occurring across health and social settings generated concern and frustration at the fragmentation and duplication of family violence response efforts. A whole of organisation approach was considered achievable if family violence was prioritised by the Ministry of Health as a health issue. However, an approach had to recognise that different health care settings would require different implementation strategies. The unique nature of primary health care was frequently expressed with both PHOs and general practices holding strong beliefs that primary health care was different to hospital-based care provided by a DHB. This difference was often made visible when primary health care providers attended the DHB VIP training. “Staff went and did the DHB VIP training, came back with the realisation that actually it didn’t strictly apply to primary care. It was completely different in primary care” (Family Violence Intervention Coordinator). While joining response efforts with others was perceived as beneficial, collaboration also held risk for response autonomy. Sites felt it important to maintain autonomy over the response and not be restricted by another organisation’s approach and resources. A local response built on key local relationships which met the needs of the local context and population was highly valued by focus group participants. Interestingly, local autonomy was not identified within the Network data.

The primary concern for the future was to establish recognition and support for addressing family violence as a health issue within primary health care nationally. This was considered achievable by prioritising family violence as a target health issue and providing support to implement a comprehensive response to family violence. High level system support would allow practices to feel confident in their organisation as a place where the community, and victims of family violence, knew they could receive safe care.

*Practices, we’ve got them geared up, we’ve got them thinking, we’ve created that awareness, and we don’t have further support. What happens then? We would lose the momentum we’ve created…If once you start this and you stop somewhere in the middle and you don’t continue education and support, you’ve lost the GP practice and in saying that you’ve lost the patient* (Clinical Facilitator).

## Discussion

Implementing a response to family violence within the primary health care setting is, at best, challenging. While several New Zealand sites had progressed in providing a formal response to family violence, it was largely achieved through capitalising on unique opportunities, innovative thinking and effective relationship support. Response sustainability required the strong commitment of key people dedicated to addressing family violence and would be strengthened by prioritising family violence responsiveness as a national health target with dedicated resourcing.

Despite strong recommendations by the World Health Organisation for health care professionals to respond to family violence, limited evidence exists on how to integrate a response within health care systems [3, 4]. The New Zealand health care system began a response to family violence focused on the hospital care level. The Ministry of Health began funding VIP in 2007 following the publication of Family Violence Intervention Guidelines [19] (updated in 2016 [20]). In 2012, VIP evaluation showed hospitals had exceeded the Ministry of Health set target score for partner abuse and child abuse and neglect programme development, and the Ministry of Health set supporting integration of safety planning across primary, community and hospital care services as a VIP priority for the following 3 years [10]. VIP progress within hospitals, achieved the same year as the follow-up evaluation of primary health care sites was conducted, may have influenced sector perception on what is required to achieve similar progress within primary health care.

Nevertheless, at this time, New Zealand does not formally provide adequate policy, funding or resources for an effective national systems-led response to family violence within primary health care. Establishing a formal mandate to address family violence within primary health care was perceived to enable a comprehensive approach to family violence, ongoing funding and high level organisational support. Prioritising a response to family violence within primary health care would strengthen the commitment of key people working to develop a response and create a health care environment where avoiding family violence becomes unsustainable [21, 22]. Some argue, however, that a compulsory response may not be advisable as health care professionals who are not willing to address family violence may create increased risk for the victim’s safety and wellbeing if the response is not carried out in a sensitive caring manner [3, 21].

A study analysing intimate partner violence (IPV) integration within the Spanish health system found that health system managers highly valued structure established within government and legislation to support integration of an IPV response within health. Similar to our findings, sustainable integration was considered dependent on continuous prioritisation by political and health system decision makers over time. They also noted a lack of funding affected service delivery, particularly for new innovations like IPV [21]. Recognition of the issue by political leadership is considered an important first step in overcoming restricted funding and competing priorities [3, 21, 22]. The New Zealand experience demonstrated the challenge of developing a response to family violence without that recognition, resulting in nonlinear and highly localised response development.

Although the Primary Health Care Family Violence Responsiveness Evaluation Tool [5] provides key system elements to guide an effective response to family violence and quality improvement benchmark, further resources are needed to address how to integrate this complex intervention into a complex environment [21, 22]. Viewing the health care system as a Complex Adaptive System (CAS) can provide insight into why standard quality improvement techniques (such as implementation of the Tool) can have little effect [23]. Put briefly, the structure and form of the CAS (such as the health system response to family violence) is continually formed by the patterns of relationships between agents (such as an individual health professional or a collective general practice) and the interaction of the agents with their environment (such as the primary health care system). These interactions lead to self-organisation and the emergence of new structure and form. System change, therefore, relies on the ability of agents to self-organise through their interactions [23]. From this perspective, nonlinear and localised response development is not unexpected given the difficulties sites had in self-organising. Further analysis of the health care system from the theoretical perspective of CAS could provide new insights into effective, sustainable approaches to response development, and work towards bridging the translational gap between research and implementation.

In this paper we discussed the experiences of volunteer sites and primary health care advocates dedicated to addressing family violence as a health issue. Organisations without the strong commitment of key people would find developing a response to family violence unfeasible. Study limitations include sites (three PHOs and two general practices) declining to participate in the follow-up evaluation due to reasons of major organisational restructure and focusing efforts on MOH performance targets, reflecting the competing pressures and priorities outlined in the findings of this study. Researchers conducted thematic data analysis inductively to avoid preconceptions of an effective primary health care response to family violence. However, deductive analysis using Primary Health Care Family Violence Responsiveness Evaluation Tool [5] categories as prompts may have produced other insights into site experience. Further, as site response development is in the early stages, little is known about the routine enquiry, intervention and evaluation stages.

The findings of this study contribute to a small body of international literature which discusses responding to family violence in primary health care. Despite the distinctiveness of the New Zealand health care system, the description of the means used to overcome development challenges may be useful to others. Future research should seek to first understand the system to consider how a response might be successfully implemented across primary health care nationally and further, what may influence response sustainability.

## Conclusions

This study sought to identify factors which promote or challenge the development of a response to family violence within primary health care. We found a lack of supporting resources and prioritisation to address family violence as a health issue resulted in nonlinear and highly localised family violence response development. While the Primary Health Care Family Violence Responsiveness Evaluation Tool [5] contributes to the knowledge needed to provide an effective standardised response to family violence, and supports ongoing quality improvement, further work is needed to understand how complex health care system relationships could be utilised to support sustainable integration of a family violence response within primary health care.

## Abbreviations

CAS, complex adaptive system; DHB, District Health Board; GP, general practitioner; IPV, intimate partner violence; PHO, Primary Health Organisation; PPP, PHO Performance Programme
